# Contribution of Plasminogen Activation towards the Pathogenic Potential of Oral Streptococci

**DOI:** 10.1371/journal.pone.0013826

**Published:** 2010-11-03

**Authors:** Andreas Itzek, Christine M. Gillen, Marcus Fulde, Claudia Friedrichs, Arne C. Rodloff, Gursharan S. Chhatwal, Daniel Patric Nitsche-Schmitz

**Affiliations:** 1 Department of Medical Microbiology, Helmholtz Centre for Infection Research, Braunschweig, Germany; 2 Institute for Medical Microbiology and Epidemiology of Infectious Diseases, University of Leipzig, Leipzig, Germany; Duke University Medical Center, United States of America

## Abstract

Oral streptococci are a heterogeneous group of human commensals, with a potential to cause serious infections. Activation of plasminogen has been shown to increase the virulence of typical human pathogenic streptococci such as *S. pneumoniae*. One important factor for plasminogen activation is the streptococcal α-enolase. Here we report that plasminogen activation is also common in oral streptococci species involved in clinical infection and that it depends on the action of human plasminogen activators. The ability to activate plasminogen did not require full conservation of the internal plasminogen binding sequence motif FYDKERKVY of α-enolase that was previously described as crucial for increased plasminogen binding, activation and virulence. Instead, experiments with recombinant α-enolase variants indicate that the naturally occurring variations do not impair plasminogen binding. In spite of these variations in the internal plasminogen binding motif oral streptococci showed similar activation of plasminogen. We conclude that the pathomechanism of plasminogen activation is conserved in oral streptococci that cause infections in human. This may contribute to their opportunistic pathogenic character that is unfurled in certain niches.

## Introduction

Oral streptococci are commensal microorganisms that may exert beneficial probiotic effects when residing at their natural habitat, the oral cavity. However, when they gain access to deeper oral tissues, the microorganisms invoke pathogenic processes in order to ensure their own survival, thereby becoming a substantial threat to the host with whom they normally live in symbiosis. Oral streptococci of the anginosus- and the mitis group are frequently found in human infections. The anginosus group (*Streptococcus anginosus*, *S. constellatus*, *S. intermedius*) is associated with bacteremia, purulent infections and severe abscess formation in the deep neck and in inner organs [1], [2], [3], [4], with *S. constellatus* also implicated in the pathogenesis of periodontitis [5]. Systemic infections with oral streptococci, in particular with members of the mitis group (*S. mitis*, *S. oralis*, *S. gordonii*, *S. sanguinis*, *S. parasanguinis*), are a very frequent cause for infective endocarditis [6], [7]. Despite considerable pathogenic potential of the causative organisms and the clinical relevance of infections with oral streptococci, their molecular pathogenesis is barely understood. Some of their pathogenic actions may be unique to oral streptococci, while others may show high similarity to those of the more extensively studied streptococci. The latter very often use the host plasminogen system in order to invade tissues and establish an infection. The physiological role of the plasminogen system is the degradation of fibrin clots during wound healing. In non-infected tissue the zymogen (plasminogen) is proteolytically activated to plasmin by tissue type plasminogen activator or urokinase. The activated enzyme plasmin is a broad-spectrum serine protease which degrades not only fibrin, but also extracellular matrix proteins such as fibronectin, laminin and proteoglycans. The protease inhibitors α_2_-antiplasmin and α_2_-macroglobulin tightly control the activity of plasmin [8]. Bacteria exploit the proteolytic activity of the plasminogen system to overcome physical barriers formed by the host's extracellular matrix and the coagulation system when invading host tissue; a prerequisite for successful infection and bacterial dissemination (reviewed in [8]). Streptococci have developed a variety of factors and mechanisms for surface capture and activation of plasmin(ogen). Some streptococci exploit the host's plasminogen activators [9], [10], [11]. *S. pyogenes* and species that belong to the Lancefield groups C and G express their own potent plasminogen activator, streptokinase, which changes the conformation of the unprocessed catalytic domain of plasminogen into a proteolytically active form [8]. In addition, binding to streptococcal plasminogen receptors may prevent the action of host inhibitors as seen for α_2_-antiplasmin and the broad spectrum protease inhibitor α_2_-macroglobulin [12], [13]. M-like proteins such as PAM, Prp, MLC36 and MLG72 confer the ability to bind high quantities of plasmin(ogen) to *S. pyogenes* [9], [14] or group C and G streptococci [15], thereby increasing their virulence [16]. Interestingly strains of *S. pyogenes* and *S. pneumoniae* that do not possess specialized plasminogen receptors make use of their own metabolic enzymes to recruit plasmin(ogen). Glyceraldehyde-3-phosphate dehydrogenase (GAPDH) and α-enolase, which normally are located intracellularly, act as plasmin(ogen) receptors on the surface of both *S. pyogenes* [17], [18], [19] and *S. pneumoniae* [20], [21]. The α-enolase of *S. pneumoniae* has been implicated in plasmin-dependent penetration of biological membranes during invasive infections [22]. The efficiency of plasminogen activation by α-enolase and the resulting invasiveness depends not only on two lysine residues in the C-terminal end of the streptococcal protein [23], but is also mediated by an internal binding motif: FYDKERKVY [22], [24]. Recent investigations on the α-enolase of *S. mutans* [25] suggest that variations of this internal binding motif exist (FYDNG—VY), which may impair efficient plasminogen binding and activation. Here we describe the natural variations that occur within the internal plasminogen binding motif of streptococcal α-enolase from different species. Moreover, we have comprehensively investigated the influence of natural variations on plasminogen binding and overall activation of plasminogen by oral streptococci.

## Results

### Binding of plasminogen to Streptococcus oralis

**Figure 1:**
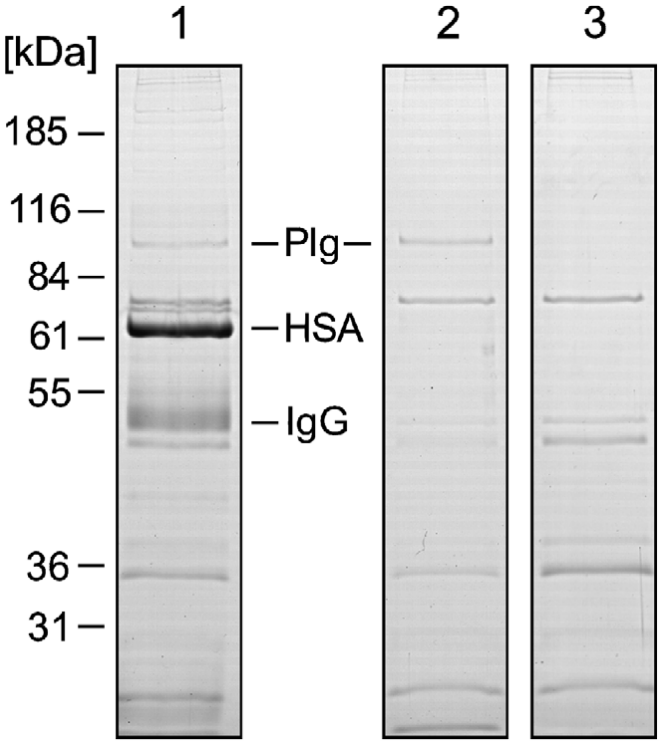
Binding of human plasma proteins to *Streptococcus oralis*. After incubation of *Streptococcus oralis* SV11 with human blood plasma (*1*), PBS containing purified human plasminogen (*2*), or PBS alone (*3*) proteins that were bound to the bacterial surface were eluted with glycine buffer (pH 2) and separated by 12% SDS-PAGE under reducing conditions. The proteins were stained with Coomassie Brilliant Blue. Mobility of marker proteins is indicated at the left and their molecular mass is given in kDa. Protein bands that were identified as plasminogen (*Plg*), human serum albumin (*HSA*), and immunoglobulin G (*IgG*) are marked. The control experiment (*3*) indicates proteins of bacterial origin.

Streptococci are known to interact with various plasma proteins. Such multiple interactions may influence one another by masking or competing for binding sites and by providing new binding sites for secondary interactions. Therefore, the interactions of the clinical *S. oralis* isolate SV11 with human plasma proteins were investigated by incubating the bacteria with human plasma or PBS as a control. Bound proteins were eluted from the surface of the bacteria, separated by SDS-polyacrylamide gel electrophoresis (SDS-PAGE) (Fig. 1), and identified by either MALDI-TOF-MS or by immunoblot analysis (data not shown). This experiment identified three major ligands bound by *S. oralis* isolate SV11: serum albumin, IgG, and plasminogen. An analogous experiment, in which *S. oralis* isolate SV11 was incubated with a solution of purified human plasminogen demonstrated its direct interaction with the bacterial surface indicating that the binding does not depend on other factors from human plasma (Fig. 1).

### Surface localization of α-enolase on S. oralis

**Figure 2:**
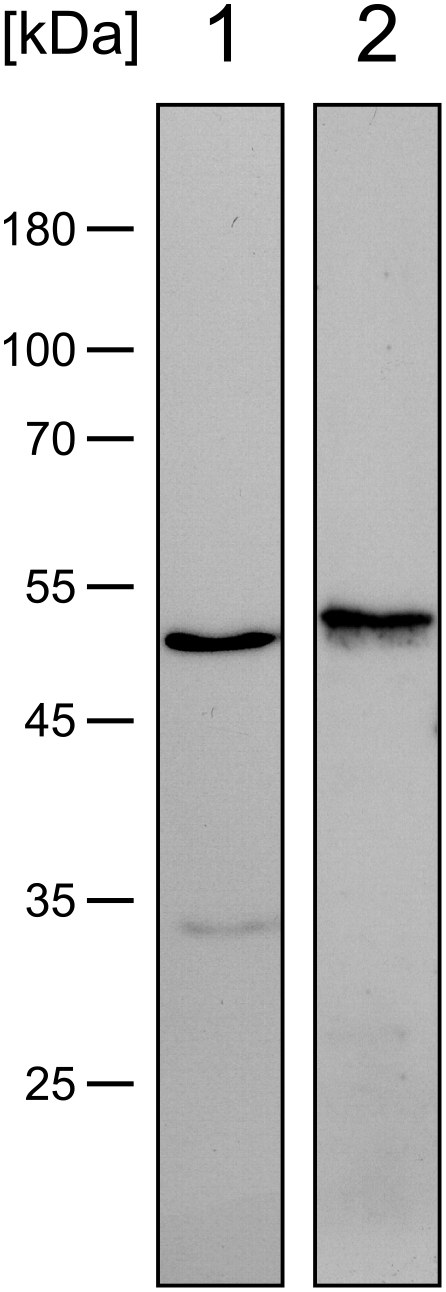
Bacterial α-enolase and GAPDH in bacterial surface eluates of *Streptococcus oralis*. Surface bound proteins of *S. oralis* SV11 were eluted with sodium carbonate buffer (pH 10), separated by 12% SDS-PAGE under reducing conditions and analysed by immunoblot with rabbit antisera specific for α-enolase (*1*) or GAPDH (*2*), respectively. Mobility of marker proteins is indicated at the left and their molecular mass is given in kDa.

Examination of alkaline eluates of streptococcal surface molecules by immuno blot with anti-sera specific for α-enolase and GAPDH (Fig. 2) indicated the presence of two proteins of *S. oralis* SV11 that are known plasminogen binding factors in other streptococcal species [17], [18], [20], [21]. Specificity of this experiment was supported by the observation that the mobility of the proteins in SDS-PAGE, which corresponded to molecular masses of 51 and 53 kDa, respectively, was in agreement with the calculated molecular masses of streptococcal α-enolase (47 kDa, GenBank accession no.: ZP_06612198) and GAPDH (36 kDa to 50 kDa; GenBank accession no.: BAD02480 and ZP_06612207). The experiment demonstrated the presence of two non-covalently bound plasminogen binding factors on the surface of *S. oralis* SV11. It remained to be tested if plasmin(ogen) binding to the surface of the bacteria were concomitant with activation of the protease.

### Plasminogen activation by S. oralis

**Figure 3:**
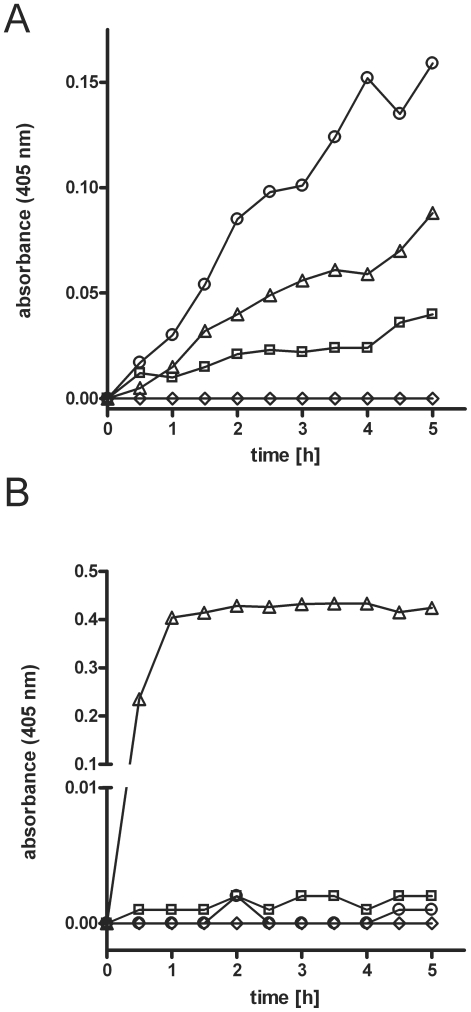
Plasminogen activation by *Streptococcus oralis*. (*A*) Bacterial cells from mid-logarithmic culture of *S. oralis* SV11 were incubated in human plasma (**○**) or PBS (**⋄**). Plasma without bacteria was used as a control for auto-activation (**□**). Incubation of plasma with 100 ng urokinase served as a positive control (**▵**) (B) Bacterial cells from mid-logarithmic culture of *Streptococcus oralis* SV11 were incubated in PBS (**⋄**) or PBS containing purified human plasminogen (**○**). Plasminogen without bacterial cells (**□**) served as a control for auto-activation. Incubation of human plasminogen with 100 ng urokinase served as a positive control (**▵**), demonstrating that the plasminogen could be activated. Plasminogen activation was measured using the colour reaction of plasmin-specific substrate (S-2251). The diagrams depict the absorbance of the liquid phase measured at a wavelength of 405 nm vs. time.

In human plasma, plasminogen activation is tightly controlled by potent inhibitors, α_2_-antiplasmin and α_2_-macroglobulin. To test the ability of *S. oralis* to generate plasmin activity against the action of the inhibitors, experiments with a plasmin(ogen) specific substrate (S-2251) were conducted (Fig. 3A). Controls demonstrated that human plasma or the SV11 strain in PBS, possessed low or no intrinsic activity against the S-2251 substrate, respectively. Incubation of human plasma with *S. oralis* SV11, as compared to the controls, led to a strongly increased conversion of the substrate after 90 min that continued for up to 6.5 h, which demonstrated that *S. oralis* SV11 can activate plasminogen against the actions of its inhibitors in human plasma (Fig. 3A). In additional experiments, *S. oralis* SV11 when incubated with purified plasminogen in PBS failed to cause substrate conversion, indicating a lack of a streptococcal plasminogen activator and thus activation by host factors from plasma (Fig. 3B).

### Variations in the internal plasmin(ogen) binding motif of α-enolase in clinical isolates of oral streptococci

Previous work on *S. pneumoniae* α-enolase revealed that plasmin(ogen) binding is mediated not only by two C-terminal lysine residues, but also by an internal plasmin(ogen) binding motif (IPM) with the sequence FYDKERKVY, which has a crucial influence on the efficiency of plasmin(ogen) binding and utilization. The distribution, conservation and species specificity of the IPM within the streptococci had not been comprehensively studied. Consequently, the knowledge about its general role in streptococcal pathogenesis remained insufficient. Sequencing of the enolase gene (*eno*) revealed that strain *S. oralis* SV11 had an α-enolase with an intact FYDKERKVY-motif. To gain insight into the distribution and conservation of the motif among the oral streptococci, a collection of 56 clinical isolates was subjected to sequence analysis of the *eno* gene (Table 1). The collection comprised the species *S. mitis, S. oralis, S. gordonii, S. sanguis* and *S. parasanguis*, which are members of the mitis group and genetically closely related to *S. pneumoniae*. Moreover, the collection consisted of strains from the anginosus group (*S. anginosus, S. constellatus, S. intermedius*) and isolates of *S. salivarius*. An intact FYDKERKVY motif was present in 46 of the 56 strains and was found in all of the examined species. Of the nine *S. salivarius* isolates examined six strains had an IPM-like motif FYDAERKVY which was also found in the majority of *S. agalactiae* (group B streptococci; i.e. GenBank accession numbers: ABA45999.1, AAM99524.1, CAD46252.1) and *S. thermophilus* (i.e. GenBank accession numbers: ABJ65942.1, AAV62231.1, AAV60341.1) strains. In *S. salivarius* SV32 the FYDKERKVY-motif was conserved, while two strains (*S. salivarius* SV17 and SV107) possessed an α-enolase with a variant FYENG—VY-motif, which resembles the IPM-like sequences in the cariogenic streptococci *S. mutans* (FYDNG—VY) and *S. sobrinus* (FYEDG—VY). In all but two of the strains that belong to the mitis group the prototypical IPM of *S. pneumoniae* was conserved. In one of the six *S. mitis* and one of the seven *S. oralis* isolates the α-enolase contained a FYDKERQVY-motif. Interestingly the IPM of *S. pneumoniae* was also present in the less related (based on comparison of 16S-rRNA gene sequence [26]) streptococci of the anginosus group. The previously described C-terminal plasminogen binding motif, which is formed by two terminal lysine residues (KK) [23], [24], [27], was conserved in 34 of the 56 isolates, this excludes all 19 anginosus group strains and a *S*. *salivarius* strain (SV121) which all possessed a terminal SK instead. In all the strains with the SK variant the FYDKERKVY sequence of the IPM was conserved. In summary, the comparison of the IPM(-like) amino acid sequences revealed a lack of species specificity. Moreover, a high prevalence of the FYDKERKVY sequence in clinical isolates of different and distantly related oral streptococci suggested that a conserved IPM may be of advantage for streptococci.

**Table 1:** Internal plasminogen binding motif (IPM) of α-enolase of oral streptococci.

Group	(Sub-)Species	No. of Isolates	Strains	IPM
Anginosus group	*S. anginosus*	8	SV29, SV33, SV38, SV52, SV66, SV75, SV91, SV92	FYDKERKVY
	*S. constellatus ssp. constellatus*	5	SV21, SV94, SV95, SV97, SV99	FYDKERKVY
	*S. constellatus ssp. pharyngis*	2	SV35, SV102	FYDKERKVY
	*S. intermedius*	4	SV101, SV103, SV105, SV106	FYDKERKVY
				
Mitis group	*S. gordonii*	2	SV55, SV118	FYDKERKVY
	*S. mitis*	1	SV28	FYDKER**Q**VY
		5	SV111, SV112, SV113, SV114, SV115	FYDKERKVY
	*S. oralis*	1	SV18	FYDKER**Q**VY
		6	SV11, SV12, SV51, SV54, SV116, SV117	FYDKERKVY
	*S. parasanguinis*	6	SV13, SV50, SV126 SV127, SV132, SV134	FYDKERKVY
	*S. sanguinis*	7	SV20, SV73, SV119, SV121, SV122, SV123, SV125	FYDKERKVY
				
Salivarius group	*S. salivarius*	2	SV17, SV107	FY**ENG**—VY
		1	SV32	FYDKERKVY
		6	SV37, SV41, SV69, SV87, SV89, SV109	FYD**A**ERKVY

### Plasminogen binding of streptococcal α-enolases with IPM variants

**Figure 4:**
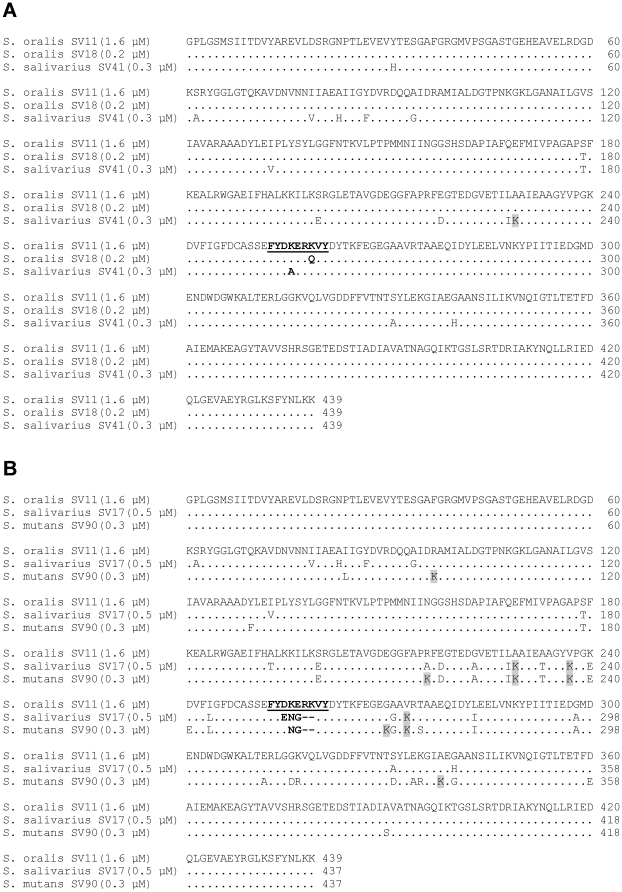
Alignment of recombinant α-enolase variants. Proteins sequences of recombinant α-enolases originating from oral streptococcal strains (A) SV11, SV18, SV41 and from (B) SV11, SV17, SV90 were aligned using CLUSTALW 2.0.12. Streptococcal species and strain designation are indicated at the left, followed by the apparent dissociation constant for plasminogen interaction in brackets (see also: Fig. 5). Dots indicate amino acids that are identical in the upper sequence. Substitutions by lysine are highlighted in gray. The IPM and its variants are depicted in bold.

**Figure 5:**
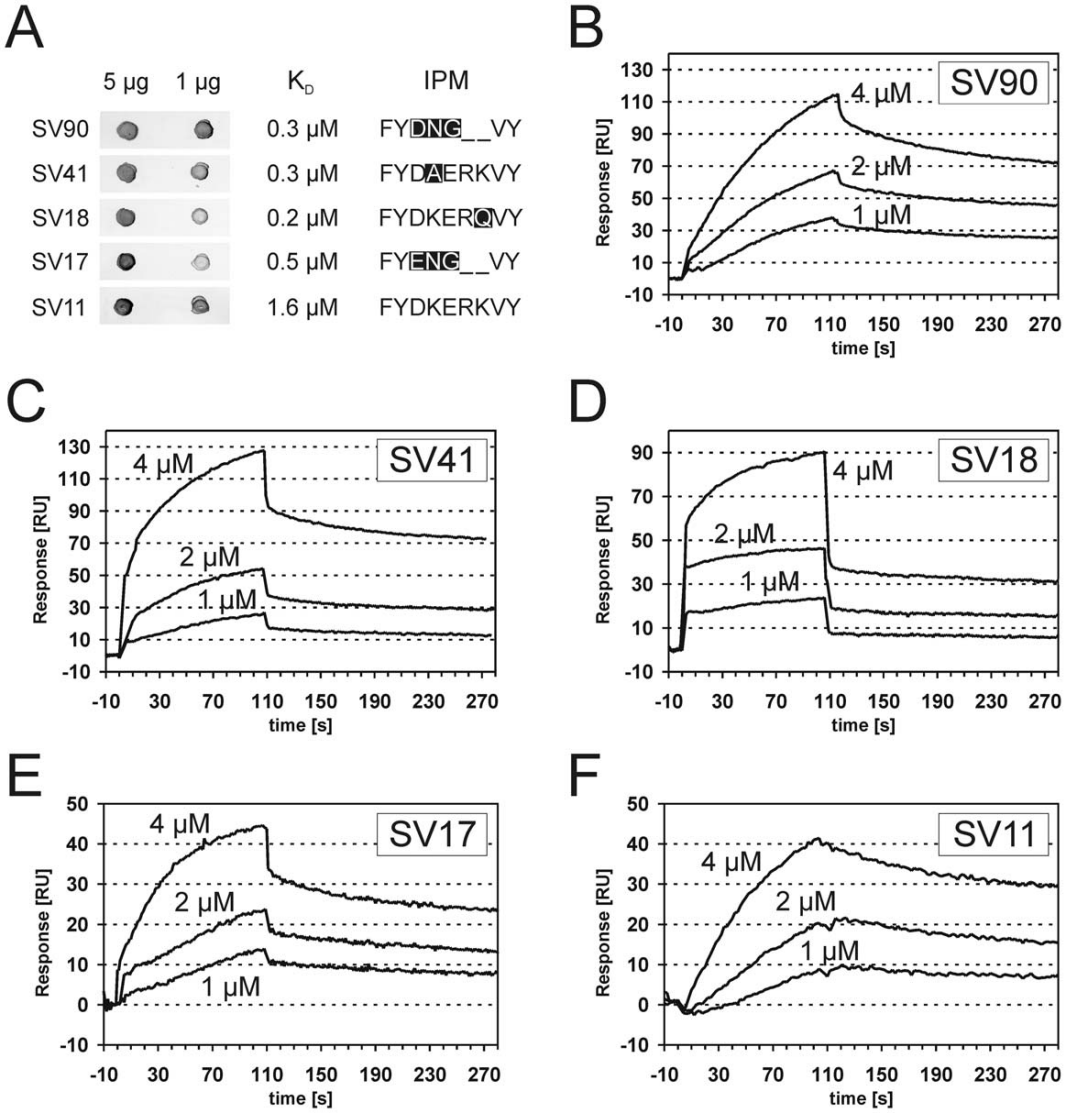
Interaction between human plasminogen and recombinant α-enolase variants. Binding was analyzed in a ligand blot experiment (A) with immobilized recombinant α-enolase variants (5 µg and 1 µg) and human plasminogen as soluble analyte. Designation of the original oral streptococcal strain and the IPM are given together with the apparent dissociation constants, which where determined by surface plasmon resonance measurements (B–F) at different analyte concentrations (4 µM, 2 µM and 1 µM α-enolase). Although curve shapes suggested interactions of higher complexity, apparent dissociation constants were determined based on the Langmuir model for 1∶1 interaction.

The variations in the IPM may influence the interaction between plasmin(ogen) and α-enolase. Therefore, five different α-enolases that represent all the sequence variations in the internal plasminogen binding motif encountered in the collection of oral streptococci were recombinantly expressed and isolated (Fig. 4). All recombinant proteins carried the two C-terminal lysine residues that are known to contribute to the interaction with plasminogen [24], [27] (Fig. 4). Binding experiments using dot-blots and surface plasmon resonance measurements delivered consistent results. All variants of the α-enolase interacted with radiolabeled human plasminogen in dot blots, generating signals of similar intensity which were comparable to α-enolase with the originally described IPM (Fig. 5). Concentration dependent binding of all α-enolase variants to immobilized human plasminogen was observed in surface plasmon resonance measurements, with similar apparent dissociations constants, ranging from 1.6 µM to 0.2 µM (Fig. 5). Conclusively, naturally occurring variations in the IPM, as encountered in oral streptococci, did not impair plasminogen binding.

### Comparison of plasminogen activation on different species of oral streptococci

**Figure 6:**
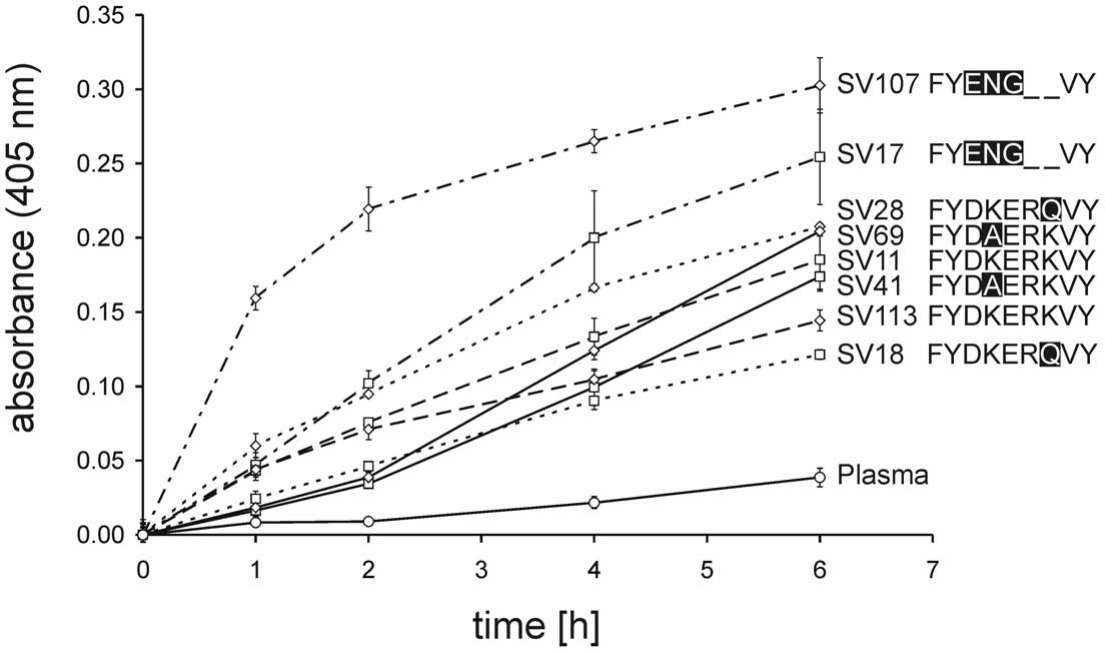
Comparison of plasminogen activation in human blood plasma by oral streptococci. Bacterial cells from mid-logarithmic culture of eight different oral streptococci strains with 4 different IPMs (Table 1) were incubated in human blood plasma (**□** and **⋄**). Plasma without bacterial cells (**○**) served as a control. Plasminogen activation was measured using the colour reaction of plasmin-specific substrate (S-2251). Absorbance of the liquid phase was measured over time at a wavelength of 405 nm. Bars represent the standard deviation of the experimental triplicates.

Differences in plasminogen activation by streptococci may crucially influence the pathogenesis of infection. Therefore, the overall plasminogen activation in human plasma by oral streptococci with different naturally occurring variants of the IPM was compared (Fig. 6). All of the streptococcal isolates led to an increased substrate conversion reflecting increased plasminogen activation as compared to a negative control without bacteria. Considerable differences in the activation were observed between different strains although without clear correlation with the IPM sequence or other mutations in the protein sequence. Only the two *S. salivarius* strains SV17 and SV107 that contain a FYENG—VY-motif showed a notably faster activation of plasminogen as compared to the other strains. Instead, functional conservation between different variants of the IPM, as demonstrated in the experiments described above, suggests that this effect depends either on quantitative differences in surface bound α-enolase or on other streptococcal factors that activate plasminogen. In all tested strains the C-terminal 70 amino acids of α-enolase were identical and terminated with two lysine residues (GenBank accession numbers: HQ398283, HQ398286, HQ398289, HQ398290, HQ398309, HQ398310, HQ398313, HQ398314). Thus, diversity in the C-terminal plasminogen binding motif was not responsible for the observed differences in plasminogen activation. Taken together, our experiments have demonstrated a similar capability of different oral streptococci species that can cause disease in humans to activate plasminogen independent of the naturally occurring variations in the previously reported IPM with the sequence FYDKERKVY [22].

## Discussion

Degradation of host proteins by generation of plasmin-activity is considered beneficial for streptococcal survival and dissemination of infection by providing bacterial nutrition and increasing bacterial mobility in the infected tissue [8], [22], [28], [29]. The experiments with *S. oralis* demonstrated that binding of plasminogen to the oral streptococci leads to its activation in a process that fully depends on plasminogen activators of the host, contrary to *S. pyogenes* and some of the Group C and G streptococcal species, which express their own bacterial plasminogen activator streptokinase [30], [31]. This is consistent with the fact that homologues of streptokinase genes were not found in the whole genome sequences that were publicly available for oral streptococci and several *S. pneumoniae* strains http://www.ncbi.nlm.nih.gov/genomes/MICROBES/microbial_taxtree.html.

Previous work on *S. pyogenes* and *S. pneumoniae* has demonstrated that the streptococcal intracellular metabolic enzymes, α-enolase and glyceraldehyde-3-phosphate dehydrogenase (GAPDH), are actively involved in binding to plasminogen triggering its activation by the plasminogen activators of the host [17], [18], [20], [21], [23], [24]. The importance of the internal plasminogen binding motif of α-enolase (IPM) for this virulence related function on the surface of *S. pneumoniae* has been demonstrated unequivocally in experiments with synthetic peptides, recombinant protein variants and mutated bacterial strains [22], [24]. Mutations in the lysine residues and the glutamic acid residue of the FYDKERKVY motif abolish plasminogen binding [24], thereby drastically diminishing the α-enolase driven activation of plasminogen by host plasminogen activators [22] and the recruitment of plasmin(ogen) to the bacterial surface [24]. As a consequence degradation of extracellular matrix and fibrin clots by the *S. pneumoniae* mutant is impaired [22]. The significant role of the IPM in *S. pneumoniae* pathogenesis is indicated by the attenuated virulence of the mutant strain in intranasally infected mice [24]. *S. pneumoniae* is genetically closely related to the oral streptococci of the mitis group. Interestingly, cross species comparison of streptococcal α-enolase revealed that the FYDKERKVY motif of *S. pneumoniae* is not only conserved in the clinically important human pathogen *S. pyogenes*, but also in the oral streptococci species, which are considered as limited in their propensity to cause disease. As shown in this study (Table 1), the FYDKERKVY motif is conserved in the α-enolases of the anginosus group (19 isolates) and mitis group (26 isolates) streptococci with two exceptions, one *S. oralis* (SV18) and one *S. mitis* isolate (SV28). The majority of *S. salivarius* strains (8 out of 9) possess variants of the IPM. According to experiments with isolated recombinant α-enolases none of the naturally occurring variations in the IPM impaired plasminogen binding, a reduction in which may diminish their ability to drive plasminogen activation by the responsible host factors.

The mode of the investigated interaction is not fully understood. Plasmin(ogen) is equipped with lysine binding kringle domains and its interaction with α-enolase is inhibitable by the lysine analog ε-aminocaproic acid (EACA) [22], [23], [32]. This suggested that lysine residues of the α-enolase form contacts with plasminogen kringle domains, which is in agreement with previous work that identified the C-terminal two lysines of *S. pyogenes* and *S. pneumoniae* α-enolase as a plasminogen binding motif [23], [24], [27]. Successive deletion of the two terminal amino acids led to a gradual decrease in plasminogen affinity, indicating that a single terminal lysine is sufficient to strengthen the interaction [23]. However, replacement of the terminal KK by KL or by LL led to a similar reduction in plasminogen affinity in both mutated proteins [23], [24]. This suggested that the terminal of the two lysine residues had a more important role in the interaction between α-enolase and plasminogen. Notably, the KL and the LL variant showed a lower affinity than the KK deletion mutant [23], [24], [27]. Thus, in addition to a potential role as a contact site for plasminogen, the terminal lysine appears to exert a stabilizing function on the α-enolase structure that is important for binding of plasminogen via the IPM. In our study the terminal KK motif was conserved in 64% of the oral streptococcal isolates. The remaining 36%, all anginosus group strains and one *S. sanguinis* strain, terminated with SK. Based on the data described above, one could speculate that the penultimate position could tolerate such substitution of the lysine residue without strong detrimental effect on plasminogen binding. However, the interaction between plasminogen and α-enolases with the C-terminal SK plasminogen remains to be studied. In contrast to several other lysine substitutions in the α-enolase [23], [27], substitution of K^344^ by glutamic acid had a destabilizing effect on the ternary structure of *S. pyogenes* α-enolase, which increased plasminogen binding but abolished phosphopyruvate hydratase activity [27]. As expected for α-enolases of viable bacteria, the analogous residue was conserved in all the examined strains of our study.

Amino acid substitutions in the IPM of α-enolase that targeted its lysine residues abolished plasminogen binding (FYD**LG**R**L**VY [22], [24]; FYD**A**ER**A**VY [27]). Alanine substitution of both lysine residues of the IPM induced limited structural changes that did not affect the ternary structure of the protein or its enzymatic activity [27]. In light of the inhibitory effect of EACA on the interaction [22], [23], [32], it seemed conceivable that the lysine residues of the IPM were directly involved in plasminogen binding, by forming contact with lysine binding sites in the host protease. However, it cannot be excluded that conformational changes in plasminogen upon EACA binding may be the reason for the inhibition rather than a competition for the lysine binding site [33], [34], [35], [36], since they could affect a possible lysine-independent binding motif of the protein. Our comparison of α-enolase variants from different viable streptococcal strains demonstrated a retained plasminogen binding activity in spite of deviations from the previously described IPM, which comprise the loss of lysine residues. In the case of α-enolase from SV18 and SV41, substitution of K^251^ or K^254^, respectively, was associated with a moderately increased affinity for plasminogen. However, there were no compensating mutations that lead to lysine residues at other sites of the protein from SV18 (Fig. 4A). The result challenges the critical role of the IPM lysine K^251^. Only one additional lysine (K^229^) was observed in the α-enolase from SV41 (Fig. 4A) and its role in plasminogen binding remains to be tested. On the contrary, in the plasminogen binding α-enolases of SV17 and SV90, the loss of both lysine residues was accompanied by an increase in the number of lysine residues in the vicinity of the IPM (Fig. 4B), suggesting compensatory mutations and a lysine dependent mode of binding. Interestingly, strains with such α-enolases (SV17 and SV107) displayed faster plasminogen activation in assays with whole bacteria (Fig. 6). These considerations shed only limited light on the nature of the plasminogen binding, which may differ between α-enolase variants and should be resolved by structural biology techniques. However, it is possible to summarize that the ability to bind plasmin(ogen) remains conserved in oral streptococci in spite of variations in the IPM. The observed functional conservation points towards a critical role of this interaction in pathogenic as well as commensal streptococci. A previous study suggested that the surface bound plasminogen may contribute to the colonization of the oral cavity, since it facilitates adhesion to pharyngeal cells [37]. The cell adhesion function may provide additional selection pressure that has led to conservation of the plasminogen interaction in α-enolase.

Plasminogen activation depends on a multifactorial interaction with the bacterial surface. To gain a holistic view of it, we have compared plasminogen activation by oral streptococci strains with differences in the IPM of α-enolase. All strains studied generated plasmin-activity in human plasma, indicating that the naturally occurring differences in the IPM, in contrast to targeted inactivating mutations [24], do not decrease plasminogen activation. This may be due to either a conserved efficacy of the IPM, emergence of alternative binding motifs or compensating processes that comprise alternative plasminogen activating factors as well as differences in protein expression, extracellular secretion, and binding to the streptococcal surface.

In conclusion, activation of human plasminogen is common not only to typical streptococcal pathogens like *S. pneumoniae*, but also to oral streptococci. This may at least partially explain their facultative pathogenic character, which is observed in several niches of the human body.

## Materials and Methods

### Bacterial strains

Clinical isolates of oral streptococci were collected at the University Hospital Leipzig, Germany and their species was determined as described previously [38]. Bacterial strains were grown on Columbia agar containing 5% sheep blood (Becton Dickinson) or as liquid cultures inoculated from single colonies in Todd-Hewitt broth (Becton Dickinson) that was supplemented with 0.5% yeast extract (THY). The cultures were incubated at 37°C in an atmosphere with 5% CO_2_.

### SDS-Polyacrylamide Gel Electrophoresis and Immunoblots

SDS-polyacrylamide gel electrophoresis (SDS-PAGE) was performed as described by Laemmli [39]. For immunoblots, the proteins were electrophoretically transferred to nitrocellulose membranes (BioRad). Unbound nitrocellulose was saturated by incubation with blocking buffer (5% (w/v) skim milk in phosphate buffered saline pH 7.0 (PBS)) for 1 h, before the membranes were incubated with a dilution of the appropriate antiserum or antibody solution in blocking buffer. If not directly conjugated to horse radish peroxidase (HRP), bound antibodies were detected using peroxidase-conjugated swine anti-rabbit IgG (Dakopats). Peroxidase activity was detected by chemiluminescence using 100 mM Tris HCl, 1.25 mM 3-aminopthalhydrazide, 225 µM p-coumaric acid, 0.01% H_2_O_2_ at pH 8.8 in water and chemiluminescence films (GE Healthcare).

### Binding experiments with plasma proteins and whole bacteria

Cells from 50 ml overnight liquid culture of *S. oralis* SV11 were harvested by centrifugation for 10 min at 4000×*g*, washed twice with PBS and resuspended either in 1 ml sterile-filtered human blood plasma, 1 ml PBS or in 1 ml PBS containing 180 ng/µl purified human plasminogen (Sigma). After incubation for 1 h with shaking (1000 rpm) at 37°C the cells were harvested by centrifugation for 10 min at 16000×*g* and washed twice with PBS to remove unbound protein. Surface-bound proteins were eluted either by incubating the cells in 100 µl 0.1 M glycine pH 2.0 for 10 min at room temperature (RT) or reducing SDS-Sample buffer [39] for 5 min at 95°C. Bacterial cells were pelleted by centrifugation for 10 min at 16000×*g* and the supernatants collected. Acidic supernatants were neutralized by adding 10 µl 1 M Tris-HCl pH 9.0. The samples were analysed by SDS-PAGE (10% gel) under reducing conditions [39]. Human IgG was detected in immunoblots using polyclonal affinity purified HRP-coupled goat antibody directed against human IgG (Jackson ImmunoResearch). Plasminogen was detected using polyclonal rabbit anti-human plasminogen antibody (American Diagnostica) in combination with HRP-coupled goat anti-rabbit IgG secondary antibody.

### Mass spectrometry

Protein bands were excised from the gel after SDS-PAGE, processed for and analysed by Matrix-assisted laser desorption/ionization time-of-flight mass spectrometry (MALDI-TOF-MS) as described previously [40].

### Alkaline eluates of bacterial surface bound proteins

Bacteria from 50 ml overnight culture of *S. oralis* SV11 were washed twice in 0.1 M sodium acetate (pH 5.4). Surface bound proteins were eluted by incubating the cells in 100 µl 0.1 M sodium carbonate buffer (pH 10) for 30 min at 37°C. Bacterial cells were pelleted by 10 min centrifugation at 16000×*g*. The supernatants were analyzed by 10% SDS-PAGE and immunoblots with rabbit-raised antisera specific for streptococcal α-enolase [21] or GAPDH [20], respectively.

### Plasminogen activation by whole bacteria and by recombinant α-enolase

Overnight cultures of the bacteria were diluted 1∶30 in 50 ml THY and grown until mid-logarithmic growth-phase (OD_600 nm_ = 0.6). Bacteria were harvested by centrifugation for 10 min at 4000×*g* and washed twice with PBS before they were resuspended in 1 ml filtered (0.2 µm) human blood plasma or PBS. When required, 100 ng urokinase (Sigma-Aldrich) was added. Samples without bacteria were used as controls. To monitor plasminogen activation, the plasmin-specific chromogenic substrate S-2251 (Chromogenix) was added to a final concentration of 400 µM and the samples were incubated for 6.5 h while shaking (1000 rpm) at 37°C. At 30 min time intervals, 100 µl samples were taken and centrifuged for 5 min at 16000×*g* before the absorbance of the supernatant was measured at a wavelength of 405 nm in an ELISA-reader (Tecan). Analogously, the plasminogen activation by α-enolase was studied using the isolated recombinant proteins (1 µg) instead of the bacteria.

### Sequencing of the eno-gene

Genomic DNA was isolated from 15 ml overnight culture of oral streptococci using the DNeasy® Blood & Tissue Kit (Qiagen) with minor modification of the manufacturer's protocol. Bacteria were washed twice with PBS and lysed by incubation for 30 min at 37°C in 460 µl lysis-buffer (20 mM Tris-HCl, 2 mM EDTA, 1.2% TritonX-100, pH 8) containing 250 U mutanolysin (Fluka) and 50 µg RNAse A (AppliChem). Polymerase chain reaction on the streptococcal DNA with the primers Eno1 (GAC TCA CGC GGT AAC CCA AC) and Eno2 (ATT GTT GAA TCT TCA GTT TCA CC) produced 1.1 kb amplicons of internal coding sequence. For sequencing of the 3′-end the internal primer EnoLys1 (CTC AAT CCT TAT CAA AGT TAA CC) was used in combination with one of the external primers EnoLys2 (GGG CTA GAA ACG TTG TTA AAT C), EnoLys4 (GCC AGT TCT TCA ATC TTG TCC), Eno Lys6 (CTG GAT AAC GTT CAA ACA TCG) or EnoLys7 (CCA ATG GAA TTA TTT AAC CAA G). The PCR products were purified using the QIAquick PCR Purification Kit (Qiagen) and sequenced using aforementioned primers, the BigDye® Terminator v3.1 Cycle Sequencing Kit (Applied Biosystems) and an ABI Prism 377 system (Applied Biosystems). Sequence data were processed and analysed using the software BioEdit (version 7.0.8, Ibis Biosciences) and deposited in the GenBank database (GenBank accession numbers: HM751882-HM751937 and HQ389262-HQ389317).

### Cloning, overexpression and purification of recombinant α-enolase

The *eno* genes were amplified from chromosomal DNA using forward and reverse primers incorporating a *BamHI* or *SalI* restriction site, respectively (primer 1: GCG GAT CCA TGT CAA TTA TTA CTG ATG TTT ACG, primer 2: GCT GTC GAC TTA TTA TTT TTT AAG GTT GTA GAA TGA TTT). Amplified *eno* genes were cloned into the multiple cloning site of the pGEX-6P vector (GE Healthcare), according to manufacturer's instructions, resulting in a fusion protein with an N-terminal glutathione *S*-transferase (GST) tag. Recombinant α-enolase was overexpressed and purified, according to manufacturer's instructions, from 500 ml *E. coli* HB101 cultures, using Glutathione Sepharose™ 4 Fast Flow (GE Healthcare) affinity chromatography. The recombinant α-enolase was cleaved from the GST-tag and released from the glutathione-sepharose column by incubation with 80 U of Precission protease per bed volume of glutathione-sepharose (GE Healthcare) in PBS overnight at 4°C. Protein eluates were dialysed against 2 l PBS overnight at 4°C. Purity of the protein fractions was analysed by SDS-PAGE (12% gel) and protein concentration was determined using bicinchoninic acid (Sigma-Aldrich).

### Surface plasmon resonance measurements

Protein interactions were studied by surface plasmon resonance measurements in a BIAcore 2000 system (BIAcore AB) as described earlier [40]. Human plasminogen (Sigma-Aldrich) was dissolved in 10 mM sodium acetate, pH 5.0 at a concentration of 500 µg/ml. Injection of 20 µl at a flow rate of 5 µl/min led to immobilization of 3500 response units (RU) of plasminogen. Residual reactive groups were inactivated by a 7 min injection of 1 M ethanolamine, 0.1 M NaHCO_3_, 0.5 M NaCl, 5 mM EDTA, pH 8.0. Surface regeneration was achieved by injection of two 30 s pulses of 0.2% SDS in water. Recombinant α-enolase as the analyte was diluted in running buffer at concentrations of 4 µM, 2 µM and 1 µM. The interaction was measured at a flow rate of 60 µl/min. The BIAevaluation 3.0 software was used for further analysis of the data. Shown curves represent the difference between the signal of the collagen-coupled surface and of a deactivated control surface devoid of protein. They were further corrected by subtraction of the curve that was obtained after injection of buffer alone. Buffer injection led to responses less than 5 RU.

### Ligand blots

Recombinant α-enolase variants were dot-blotted onto nitrocellulose (5 µg and 1 µg) before blocking with 5% (w/v) skim milk in PBS for 1 h at RT. The membrane was washed for 5 min in PBS and probed with 50 µg human plasminogen (Sigma-Aldrich, Germany) for 2 h. Then the membrane was washed 3 times for 5 min with PBS. Binding of plasminogen was detected with a rabbit raised primary antibody directed against plasminogen (Affinity Biologicals, 1∶200 in blocking buffer), followed by an HRP-conjugated secondary antibody incubation (DAKO, 1∶3000 in blocking buffer), which after washing with PBS (3 times for 5 min) was detected using 1 mg/ml 4-chlor-1-naphthol and 0.1% H_2_O_2_ in water.

## References

[1] Claridge JE, Attorri S, Musher DM, Hebert J, Dunbar S (2001). Streptococcus intermedius, Streptococcus constellatus, and Streptococcus anginosus (“Streptococcus milleri group”) are of different clinical importance and are not equally associated with abscess.. Clin Infect Dis.

[2] Jacobs JA, Pietersen HG, Stobberingh EE, Soeters PB (1994). Bacteremia involving the “Streptococcus milleri” group: analysis of 19 cases.. Clin Infect Dis.

[3] Whiley RA, Beighton D, Winstanley TG, Fraser HY, Hardie JM (1992). Streptococcus intermedius, Streptococcus constellatus, and Streptococcus anginosus (the Streptococcus milleri group): association with different body sites and clinical infections.. J Clin Microbiol.

[4] Ruoff KL (1988). Streptococcus anginosus (“Streptococcus milleri”): the unrecognized pathogen.. Clin Microbiol Rev.

[5] Colombo AP, Haffajee AD, Dewhirst FE, Paster BJ, Smith CM (1998). Clinical and microbiological features of refractory periodontitis subjects.. J Clin Periodontol.

[6] Douglas CW, Heath J, Hampton KK, Preston FE (1993). Identity of viridans streptococci isolated from cases of infective endocarditis.. J Med Microbiol.

[7] Westling K, Julander I, Ljungman P, Vondracek M, Wretlind B (2008). Identification of species of viridans group streptococci in clinical blood culture isolates by sequence analysis of the RNase P RNA gene, rnpB.. J Infect.

[8] Lähteenmäki K, Kuusela P, Korhonen TK (2001). Bacterial plasminogen activators and receptors.. FEMS Microbiol Rev.

[9] Berge A, Sjöbring U (1993). PAM, a novel plasminogen-binding protein from Streptococcus pyogenes.. J Biol Chem.

[10] Kuusela P, Ullberg M, Saksela O, Kronvall G (1992). Tissue-type plasminogen activator-mediated activation of plasminogen on the surface of group A, C, and G streptococci.. Infect Immun.

[11] Khil J, Im M, Heath A, Ringdahl U, Mundada L (2003). Plasminogen enhances virulence of group A streptococci by streptokinase-dependent and streptokinase-independent mechanisms.. J Infect Dis.

[12] Coleman JL, Benach JL (1999). Use of the plasminogen activation system by microorganisms.. J Lab Clin Med.

[13] Lottenberg R, Minning-Wenz D, Boyle MD (1994). Capturing host plasmin(ogen): a common mechanism for invasive pathogens?. Trends Microbiol.

[14] Sanderson-Smith ML, Dowton M, Ranson M, Walker MJ (2007). The plasminogen-binding group A streptococcal M protein-related protein Prp binds plasminogen via arginine and histidine residues.. J Bacteriol.

[15] Ben Nasr A, Wistedt A, Ringdahl U, Sjobring U (1994). Streptokinase activates plasminogen bound to human group C and G streptococci through M-like proteins.. Eur J Biochem.

[16] Sanderson-Smith ML, Dinkla K, Cole JN, Cork AJ, Maamary PG (2008). M protein-mediated plasminogen binding is essential for the virulence of an invasive Streptococcus pyogenes isolate.. Faseb J.

[17] Pancholi V, Fischetti VA (1998). alpha-enolase, a novel strong plasmin(ogen) binding protein on the surface of pathogenic streptococci.. J Biol Chem.

[18] Pancholi V, Fischetti VA (1993). Glyceraldehyde-3-phosphate dehydrogenase on the surface of group A streptococci is also an ADP-ribosylating enzyme.. Proc Natl Acad Sci U S A.

[19] Pancholi V, Chhatwal GS (2003). Housekeeping enzymes as virulence factors for pathogens.. Int J Med Microbiol.

[20] Bergmann S, Rohde M, Hammerschmidt S (2004). Glyceraldehyde-3-phosphate dehydrogenase of Streptococcus pneumoniae is a surface-displayed plasminogen-binding protein.. Infect Immun.

[21] Bergmann S, Rohde M, Chhatwal GS, Hammerschmidt S (2001). alpha-Enolase of Streptococcus pneumoniae is a plasmin(ogen)-binding protein displayed on the bacterial cell surface.. Mol Microbiol.

[22] Bergmann S, Rohde M, Preissner KT, Hammerschmidt S (2005). The nine residue plasminogen-binding motif of the pneumococcal enolase is the major cofactor of plasmin-mediated degradation of extracellular matrix, dissolution of fibrin and transmigration.. Thromb Haemost.

[23] Derbise A, Song YP, Parikh S, Fischetti VA, Pancholi V (2004). Role of the C-terminal lysine residues of streptococcal surface enolase in Glu- and Lys-plasminogen-binding activities of group A streptococci.. Infect Immun.

[24] Bergmann S, Wild D, Diekmann O, Frank R, Bracht D (2003). Identification of a novel plasmin(ogen)-binding motif in surface displayed alpha-enolase of Streptococcus pneumoniae.. Mol Microbiol.

[25] Jones MN, Holt RG (2007). Cloning and characterization of an alpha-enolase of the oral pathogen Streptococcus mutans that binds human plasminogen.. Biochem Biophys Res Commun.

[26] Kawamura Y, Hou XG, Sultana F, Miura H, Ezaki T (1995). Determination of 16S rRNA sequences of Streptococcus mitis and Streptococcus gordonii and phylogenetic relationships among members of the genus Streptococcus.. Int J Syst Bacteriol.

[27] Cork AJ, Jergic S, Hammerschmidt S, Kobe B, Pancholi V (2009). Defining the structural basis of human plasminogen binding by streptococcal surface enolase.. J Biol Chem.

[28] Walker MJ, McArthur JD, McKay F, Ranson M (2005). Is plasminogen deployed as a Streptococcus pyogenes virulence factor?. Trends Microbiol.

[29] Sun H, Ringdahl U, Homeister JW, Fay WP, Engleberg NC (2004). Plasminogen is a critical host pathogenicity factor for group A streptococcal infection.. Science.

[30] (1947). Studies on Streptococcal Fibrinolysis: V. The in Vitro Production of Fibrinolysin by Various Groups and Types of Beta Hemolytic Streptococci; Relationship to Antifibrinolysin Production.. J Exp Med.

[31] Christensen LR (1945). Streptococcal Fibrinolysis: A Proteolytic Reaction Due to a Serum Enzyme Activated by Streptococcal Fibrinolysin.. J Gen Physiol.

[32] Antikainen J, Kuparinen V, Lahteenmaki K, Korhonen TK (2007). Enolases from Gram-positive bacterial pathogens and commensal lactobacilli share functional similarity in virulence-associated traits.. FEMS Immunol Med Microbiol.

[33] Mangel WF, Lin BH, Ramakrishnan V (1990). Characterization of an extremely large, ligand-induced conformational change in plasminogen.. Science.

[34] Ramakrishnan V, Patthy L, Mangel WF (1991). Conformation of Lys-plasminogen and the kringle 1-3 fragment of plasminogen analyzed by small-angle neutron scattering.. Biochemistry.

[35] Marshall JM, Brown AJ, Ponting CP (1994). Conformational studies of human plasminogen and plasminogen fragments: evidence for a novel third conformation of plasminogen.. Biochemistry.

[36] Markus G, DePasquale JL, Wissler FC (1978). Quantitative determination of the binding of epsilon-aminocaproic acid to native plasminogen.. J Biol Chem.

[37] Pancholi V, Fontan P, Jin H (2003). Plasminogen-mediated group A streptococcal adherence to and pericellular invasion of human pharyngeal cells.. Microb Pathog.

[38] Friedrichs C, Rodloff AC, Chhatwal GS, Schellenberger W, Eschrich K (2007). Rapid identification of viridans streptococci by mass spectrometric discrimination.. J Clin Microbiol.

[39] Laemmli UK (1970). Cleavage of structural proteins during the assembly of the head of bacteriophage T4.. Nature.

[40] Nitsche DP, Johansson HM, Frick IM, Mörgelin M (2006). Streptococcal protein FOG, a novel matrix adhesin interacting with collagen I in vivo.. J Biol Chem.

